# Reciprocal regulation of *Yersinia pestis* biofilm formation and virulence by RovM and RovA

**DOI:** 10.1098/rsob.150198

**Published:** 2016-03-16

**Authors:** Lei Liu, Haihong Fang, Huiying Yang, Yiquan Zhang, Yanping Han, Dongsheng Zhou, Ruifu Yang

**Affiliations:** 1 State Key Laboratory of Pathogen and Biosecurity, Beijing Institute of Microbiology and Epidemiology, Beijing 100071, People's Republic of China; 2 School of Medicine, Jiangsu University, Zhenjiang 212013, Jiangsu, People's Republic of China

**Keywords:** *Yersinia pestis*, RovM, RovA, biofilm, virulence, transcriptional regulation

## Abstract

RovA is known to enhance *Yersinia pestis* virulence by directly upregulating the *psa* loci. This work presents a complex gene regulatory paradigm involving the reciprocal regulatory action of RovM and RovA on the expression of biofilm and virulence genes as well as on their own genes. RovM and RovA enhance and inhibit *Y. pestis* biofilm production, respectively, whereas RovM represses virulence in mice. RovM directly stimulates the transcription of *hmsT*, *hmsCDE* and *rovM*, while indirectly enhancing *hmsHFRS* transcription. It also indirectly represses *hmsP* transcription. By contrast, RovA directly represses *hmsT* transcription and indirectly inhibits *waaAE-coaD* transcription, while RovM inhibits *psaABC* and *psaEF* transcription by directly repressing *rovA* transcription. *rovM* expression is significantly upregulated at 26°C (the temperature of the flea gut) relative to 37°C (the warm-blooded host temperature). We speculate that upregulation of *rovM* together with downregulation of *rovA* in the flea gut would promote *Y. pestis* biofilm formation while inhibiting virulence gene expression, leading to a more transmissible infection of this pathogen in fleas. Once the bacterium shifts to a lifestyle in the warm-blooded hosts, inhibited RovM production accompanied by recovered RovA synthesis would encourage virulence factor production and inhibit biofilm gene expression.

## 1. Introduction

*Yersinia pestis*, the causative agent of plague, has caused at least three plague pandemics in human history. The transmission of bubonic and septicaemic plague in nature primarily relies on infected fleas. *Y. pestis* grows as an attached biofilm that blocks the flea's proventriculus, which enhances the flea-borne transmission of this pathogen [1].

The *Y. pestis hmsHFRS* operon is responsible for the synthesis and transport of exopolysaccharide, the primary dry component of the biofilm matrix [2]. 3,5′-cyclic diguanylic acid (c-di-GMP) is a signalling molecule that promotes biofilm exopolysaccharide production in bacteria. In *Y. pestis*, HmsT and HmsD (*hmsD* is located in the three-gene operon *hmsCDE*) are the only two diguanylate cyclases that catalyse c-di-GMP synthesis [3], whereas HmsP is the sole phosphodiesterase catalysing c-di-GMP degradation [4].

*waaA*, *waaE* and *coaD* constitute the three-gene operon *waaAE*-*coaD* in *Y. pestis*; WaaA is a 3-deoxy-d-manno-octulosonic acid transferase involved in the synthesis of lipopolysaccharide (LPS), and the deletion of *waaA* leads to a biofilm defect phenotype of *Y. pestis* [5]. The *waaAE*-*coaD* operon is necessary for the biosynthesis of integral LPS, and the truncation of LPS owing to the deletion of *waaA* in *Y. pestis* induces dramatic attenuation of virulence [6].

*Yersinia pestis psa* loci are composed of two adjacent operons: *psaABC* and *psaEF* [7]. *psaA* is the structural gene of the pH 6 antigen, a PsaA polymer fimbrial structure; PsaB and PsaC constitute a chaperone/usher machinery that mediates the secretion and assembly of pH 6 antigen on the cell surface [7]. *psaABC* expression is greatly stimulated following a rise in temperature from 26°C to 37°C and in acidic environments, whereas *psaEF* encodes the transcriptional activators of *psaABC* [8]. pH 6 antigen is an adhesin that mediates the entry of bacteria into human pulmonary epithelial cells [9,10]. It is also critical for contact of *Y. pestis* with eukaryotic cells that promotes the delivery of Yops (effectors of plasmid pCD1-encoding type III secretion system) to target host cells [11]. A further pH 6 function is as an anti-phagocytic factor, independent of Yops and the F1 capsule, which blocks bacterial uptake [12]. However, the loss of pH 6 antigen production has no effect on *Y. pestis* virulence in subcutaneously inoculated BALB/c naive mice [13]. Involvement of pH 6 antigen in *Y. pestis* virulence seems to rely on *Y. pestis* strains and animals challenged.

The ferric uptake regulator Fur controls almost all iron assimilation functions in *Y. pestis* [14], and also acts as a strong repressor of *Y. pestis* biofilm formation through direct inhibition of *hmsT* transcription [15].

*phoP* and *phoQ* can be assigned into two operons: YPO1635*-phoPQ-*YPO1632 and *phoPQ-*YPO1632 [16]. The regulator PhoP and sensor PhoQ constitute a two-component regulatory system, PhoP/PhoQ. PhoP/PhoQ production is induced in the flea gut, and is essential for the formation of flea-borne infectious *Y. pestis* biofilms [17]. PhoP has no regulatory effect on *hms* genes, but it acts as a direct transcriptional activator of *waaAE*-*coaD* [18].

RovA is required for full virulence in all three pathogenic *Yersinia* species [19–21]. *Y. pestis* RovA stimulates transcription of the CUS-2 loci [19], which encode a stably integrated prophage in the *Orientalis* biovars, but not in the other three biovars *Antiqua*, *Medievalis* and *Microtus*. This prophage is not associated with biofilm formation or flea transmission, but contributes to virulence in mice [22]. RovA also plays a critical role in modulating construction and functioning of the *Y. pestis* cell membrane [23]. Most importantly, *Y. pestis* PhoP and RovA bind to the promoter–proximal regions of *psaABC* and *psaEF* to repress and stimulate their transcription, respectively. PhoP directly represses *rovA* transcription, thereby directly and indirectly negatively regulating *psa* genes though acting on both *psa* genes and *rovA* [24,25].

RovM is a LysR-type transcription factor that directly inhibits *rovA* transcription in *Y. pseudotuberculosis*, which is genetically closely related to *Y. pestis* [26]. A set of LysR-family regulators, such as PecT in *Erwinia chrysanthemi*, HexA in *E. carotovora*, LrhA in *Escherichia coli* and YfbA in *Y. pestis* [27], were found to modulate bacterial virulence and biofilm formation [28–30]. However, it remains unknown whether RovM controls virulence and biofilm gene expression in *Y. pestis.* Similarly, an array of MarR-family regulators, such as TcaR in *Staphylococcus epidermidis*, AsrR in *Enterococcus faecium*, RcaR in *Streptococcus mutans* and SarZ/SarA in *Staphylococcus aureus*, were reported to affect biofilm formation [31–34]. RovA is another MarR-family regulator, but it is not clear whether *Y. pestis* RovA is involved in the modulation of biofilm production.

**Figure 1.**
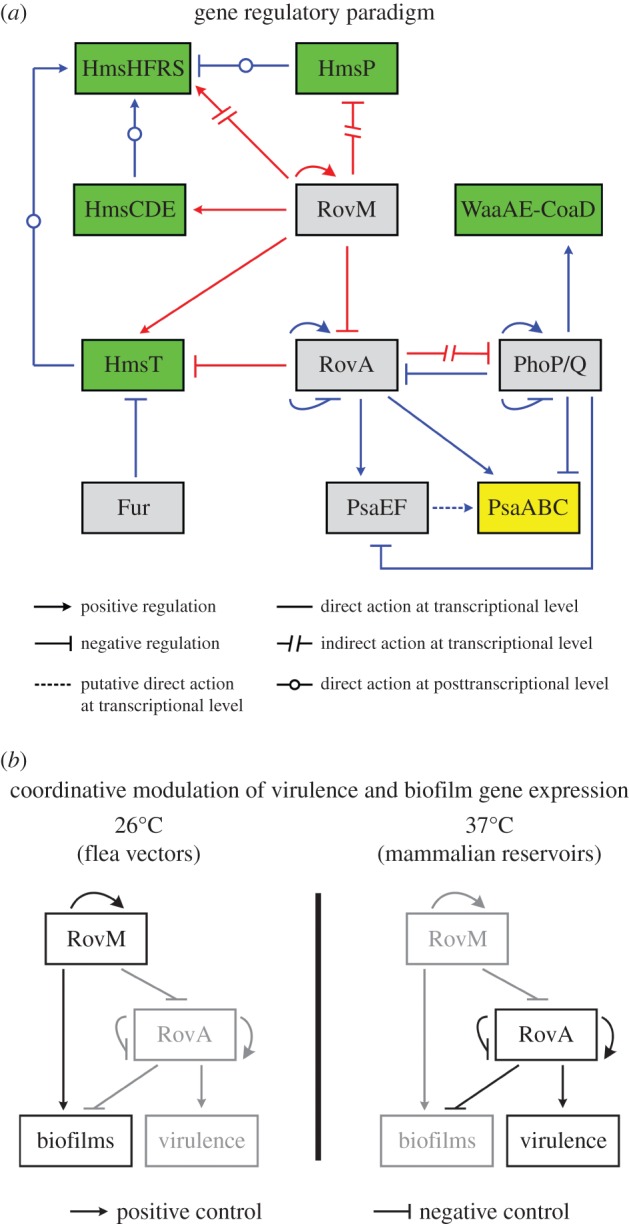
Reciprocal regulation of biofilm formation and virulence by RovM and RovA. (*a*) Gene regulatory paradigm. The gene regulatory circuits are described in the main text; those highlighted in red correspond to data presented in this work, whereas those in blue were characterized previously. (*b*) Coordinative modulation of virulence and biofilm gene expression. The proposed model of coordinative modulation of virulence and biofilm gene expression during the lifestyle shift between flea vectors and warm-blooded mammalian hosts is shown. Grey lines indicate inhibited gene expression or regulatory actions that have stopped.

This work presents a complex gene regulatory paradigm involving the reciprocal regulatory action of RovM and RovA on biofilm formation, virulence in mice and their own expression in *Y. pestis*, promoting us to gain deeper insights into coordinative modulation of virulence and biofilm gene expression during lifestyle shift between flea vectors and warm-blooded mammalian hosts (figure 1).

## 2. Results

### 2.1. RovM- and RovA-dependent biofilm and virulence phenotypes

**Figure 2.**
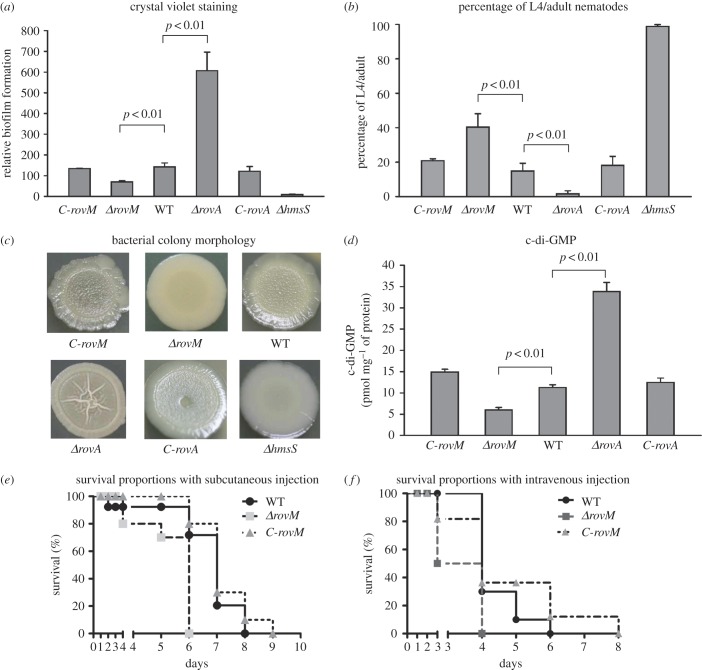
RovM- and RovA-dependent phenotypes. (*a*) Crystal violet staining of biofilms. *Y. pestis* was grown in 24-well polystyrene dishes, and the bacterial biomass adhering to the well walls was stained with crystal violet to determine the OD_570_ values. Planktonic cells were used to determine OD_620_ values. The relative capacity of biofilm formation of each strain tested was shown as the OD_570_/OD_620_ value. (*b*) Biofilms on nematodes. After the incubation of nematode eggs on the lawns of indicated *Y. pestis* strains, the nematode developmental stage on each lawn was scored to calculate the percentage of L4/adult. (*c*) Bacterial colony morphology. Aliquots of bacterial glycerol stocks were spotted onto LB plates, and incubated for one week. (*d*) Cellular c-di-GMP concentrations. Intracellular c-di-GMP concentrations were determined by high-performance liquid chromatography with tandem mass spectrometry, and values expressed as pmol mg^−1^ of bacterial protein. (*e*,*f*) Virulence in mice. BALB/c mice were challenged with approximately 300 CFU of each of WT, *ΔrovM* or *C-rovM* via s.c. (*e*) or i.v. (*f*) routes of infection.

The deletion of *rovM* induced a dramatic reduction of biofilm crystal violet staining compared with wild-type (WT) and *C-rovM*; by contrast, *ΔrovA* showed more crystal violet staining than WT and *C-rovA* (figure 2*a*). After incubation of nematode eggs on the bacterial lawns of *ΔhmsS*, 100% of the larvae grew and developed into L4/adult nematodes, whereas values for WT/*C-rovM*/*C-rovA*, *ΔrovM* and *ΔrovA* lawns were 15%, 40% and 2%, respectively (figure 2*b*). When grown on LB agar plates, *ΔrovM* colony morphology was much smoother than that of WT and *C-rovM*, whereas *ΔrovA* morphology was more rugose than that of WT and *C-rovA* (figure 2*c*). A significantly lower production of cellular c-di-GMP was observed in *ΔrovM* relative to WT and *C-rovM*, but this was much higher in *ΔrovA* compared with WT and *C-rovA* (figure 2*d*). These phenotypic results suggest that the deletion of *rovM* or *rovA* led to a dramatic reduction or elevation of biofilm/c-di-GMP production, respectively. Additionally, compared with WT and *C-rovM*, *ΔrovM* displayed a significant increase in virulence in mice after subcutaneous (s.c.) or intravenous (i.v.) injection (figure 2*e*,*f*). Our previous study showed that the deletion of *rovA* induced a significant virulence attenuation of *Y. pestis* strain 201 after s.c. or i.v*.* injection [23]. In summary, *Y. pestis* RovM and RovA appear to enhance and inhibit biofilm formation, respectively, while also inhibiting and enhancing virulence, respectively.

The first genes of the indicated multi-gene operons as well as the individual genes, which encode major biofilm determinants (*hmsT*, *hmsCDE*, *hmsHFRS*, *hmsP* and *waaAE-coaD*), the virulence factor pH 6 antigen and its local regulators (*psaABC* and *psaEF*), and a set of global regulators involved in biofilm and virulence modulation (YPO1635*-phoPQ-*YPO1632, *phoPQ-*YPO1632, *fur*, *rovA* and *rovM*), were subjected to the following gene regulation experiments to dissect the detailed regulatory action of RovM and RovA on these target genes.

### 2.2. Regulation of major biofilm genes by RovM and RovA

**Figure 3.**
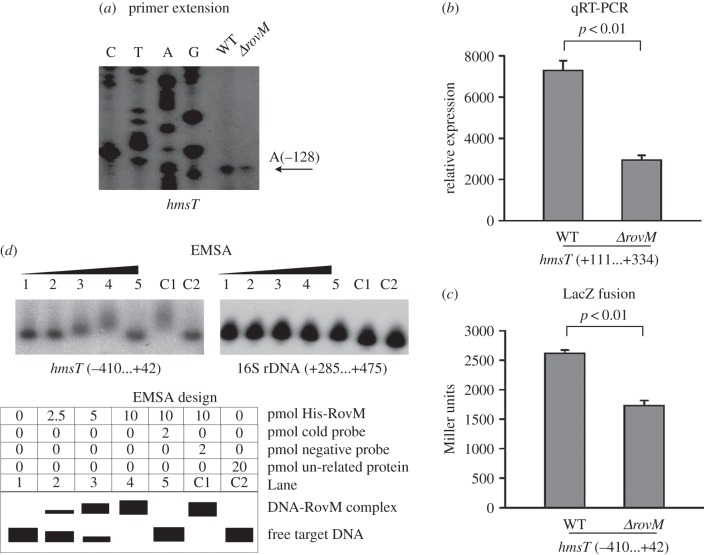
Direct activation of *hmsT* transcription by RovM. Positive and negative numbers indicate the nucleotide positions upstream and downstream of the translation start site, respectively. Lanes G, A, T and C represent Sanger sequencing reactions. (*a*) Primer extension. An oligonucleotide primer was designed to be complementary to the RNA transcript of *hmsT*. Primer extension products were analysed with an 8 M urea–6% acrylamide sequencing gel. Arrow represents the transcription start site of *hmsT*. (*b*) Quantitative RT-PCR. mRNA levels of *hmsT* were compared between *ΔrovM* and WT. A standard curve was made for each RNA preparation with the 16S rRNA gene; the relative mRNA level was determined by calculating the threshold cycle (ΔCt) of target genes via the classic ΔCt method. (*c*) LacZ fusion. A promoter–proximal region of *hmsT* was cloned into the *lacZ* transcriptional fusion vector pRW50, and transformed into WT or *ΔrovM* to determine *hmsT* promoter activity, i.e. β-galactosidase activity (Miller units), in cellular extracts. (*d*) EMSA. The radioactively labelled promoter–proximal DNA fragment of *hmsT* was incubated with increasing amounts of purified His-RovM protein, then subjected to 4% (w/v) polyacrylamide gel electrophoresis. With increasing amounts of His-RovM, the band of free target DNA disappeared, and a retarded DNA band with decreased mobility was apparent, which presumably represented the protein–DNA complex. A DNA fragment from the coding region of the 16S rRNA gene served as a negative control.

**Figure 4.**
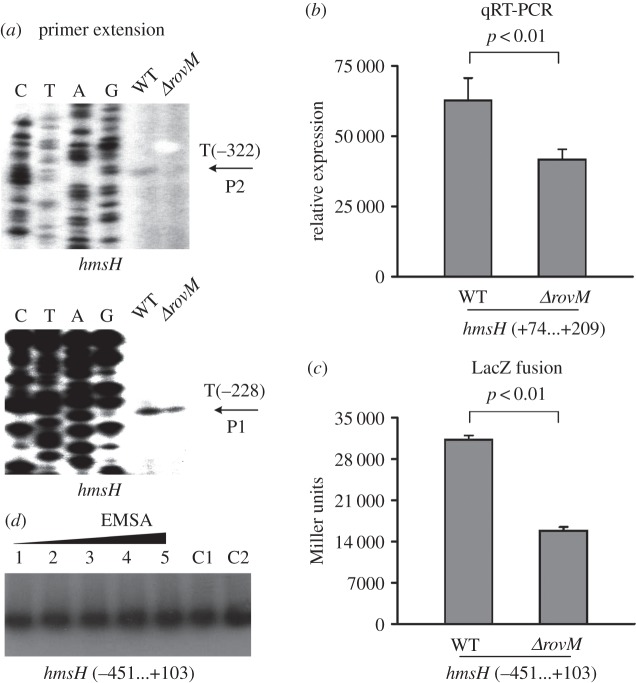
Indirect activation of *hmsH* transcription by RovM. See figure 3 for the annotations of primer extension (*a*), quantitative RT-PCR (*b*), LacZ fusion (*c*) and EMSA (*d*).

As indicated by the primer extension and real-time reverse transcriptase (RT)-PCR assays, mRNA levels of each of *hmsT* (figure 3*a*,*b*), *hmsC* (electronic supplementary material, figure S1*a,b*) and *hmsH* (figure 4*a*,*b*) were reduced in *ΔrovM* relative to WT, while that of *hmsP* (electronic supplementary material, figure S2*a,b*) was enhanced in *ΔrovM* relative to WT. The LacZ fusion assay revealed that the promoter activity of each of *hmsT* (figure 3*c*), *hmsC* (electronic supplementary material, figure S1*c*) and *hmsH* (figure 4*c*) was attenuated in *ΔrovM* relative to WT, but that of *hmsP* (electronic supplementary material, figure S2*c*) was elevated in *ΔrovM* relative to WT*.* By contrast, the primer extension, real-time RT-PCR and LacZ fusion experiments showed that RovM had no regulatory action on *waaA* at the transcriptional level (electronic supplementary material, figure S3*a*–*c*). The electrophoretic mobility shift assay (EMSA) revealed that His-RovM could bind in a dose-dependent manner to the promoter–proximal regions of *hmsT* (figure 3*d*) and *hmsC* (electronic supplementary material, figure S1*d*) but not to *hmsH* (figure 4*d*), *hmsP* (electronic supplementary material, figure S2*d*) or *waaA* (electronic supplementary material, figure S3*d*).

The primer extension, real-time RT-PCR and LacZ fusion assays also indicated that the transcription of *hmsT* (electronic supplementary material, figure S4*a*–*c*) and *waaA* (electronic supplementary material, figure S5*a*–*c*) was enhanced in *ΔrovA* relative to WT, but that the *rovA* deletion did not influence the transcription of *hmsC* (electronic supplementary material, figure S6*a*–*c*), *hmsH* (electronic supplementary material, figure S7*a–c*) or *hmsP* (electronic supplementary material, figure S8*a–c*)*.* The EMSA assay indicated that His-RovA only bound in a dose-dependent manner to the promoter–proximal region of *hmsT* (electronic supplementary material, figure S4*d*), and not to all the other target genes tested (electronic supplementary material, figure S5*d*,S6*d*,S7*d*,S8*d*).

In summary, RovM directly stimulates the transcription of *hmsT* and *hmsCDE* and also indirectly promotes *hmsHFRS* transcription; meanwhile, it indirectly represses *hmsP* transcription. RovA directly represses *hmsT* transcription and also indirectly inhibits *waaAE-coaD* transcription. Moreover, RovM has no regulatory effect on *waaAE-coaD* at the transcriptional level, and RovA does not regulate the transcription of *hmsCDE*, *hmsHFRS* or *hmsP*.

### 2.3. Regulation of virulence-related *psa* loci by RovM

The primer extension, real-time RT-PCR and LacZ fusion assays disclosed that the transcription of *psaA* (electronic supplementary material, figure S9*a*–c) and *psaE* (electronic supplementary material, figure S10*a*–*c*) was enhanced in *ΔrovM* relative to WT. The EMSA assay showed that His-RovM could not bind to the promoter–proximal regions of *psaA* (electronic supplementary material, figure S9*d*) or *psaE* (electronic supplementary material, figure S10*d*)*,* suggesting that the inhibition of *psaABC* and *psaEF* transcription by RovM is indirect.

### 2.4. Regulation of regulatory genes by RovM and RovA

The primer extension, real-time RT-PCR and LacZ fusion assays showed that the *rovM* deletion led to an elevated transcription of YPO1635 (electronic supplementary material, figure S11*a*–*c*) but had no effect on the transcription of *phoP* (electronic supplementary material, figure S12*a*–*c*) or *fur* (electronic supplementary material, figure S13*a*–*c*). The EMSA assay found that His-RovM could not bind to the promoter–proximal region of YPO1635 (electronic supplementary material, figure S11*d*), *phoP* (electronic supplementary material, figure S12*d*) or *fur* (electronic supplementary material, figure S13*d*)*.* Therefore, RovM indirectly inhibits the transcription of YPO1635*-phoPQ-*YPO1632, but it has no regulatory effect on *phoPQ-*YPO1632 or *fur* at the transcriptional level.

The primer extension assay detected three different transcriptional start sites for *rovA*, located at 78, 100 and 343 bp upstream of *rovA*, respectively; therefore, three distinct promoters (designated P1–P3, respectively) were transcribed for *rovA*. The *rovM* deletion resulted in enhanced P1 and P2 activity, but did not influence P3 activity (electronic supplementary material, figure S14*a*). The negative regulation of *rovA* by RovM was further confirmed by real-time RT-PCR (electronic supplementary material, figure S14*b*) and LacZ fusion (electronic supplementary material, figure S14*c*). The EMSA assay detected the dose-dependent binding of His-RovM to the *rovA* promoter–proximal fragment (electronic supplementary material, figure S14*d*). Therefore, RovM recognizes the *rovA* upstream DNA region in its inhibition of *rovA* transcription.

**Figure 5.**
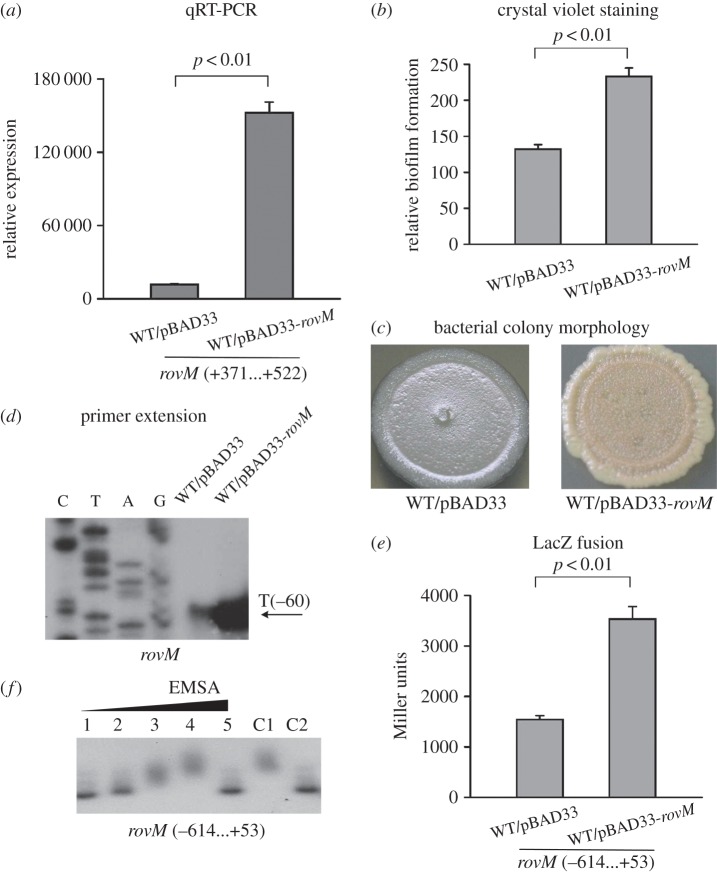
RovM upregulates its own gene*.* See figure 3 for the annotations of quantitative RT-PCR (*a*), primer extension (*d*), LacZ fusion (*e*) and EMSA (*f*). See figure 2 for the annotations of crystal violet staining of biofilms (*b*) and bacterial colony morphology (*c*).

The primer extension and LacZ fusion assays could not detect a difference in *rovM* expression between WT and *ΔrovM* (data not shown). To further characterize the potential RovM autoregulation, we constructed a *rovM*-overexpressing strain, WT*/*pBAD33-*rovM*, as well as the empty vector control strain, WT*/*pBAD33. The real-time RT-PCR assay validated the dramatically elevated mRNA expression of *rovM* in WT*/*pBAD33-*rovM* relative to WT*/*pBAD33 (figure 5*a*). Crystal violet staining and bacterial colony morphology assays further confirmed that *rovM* overexpression in WT*/*pBAD33-*rovM* led to enhanced biofilm production of this strain compared with WT*/*pBAD33 (figure 5*b*,*c*). The subsequent primer extension (figure 5*d*) and LacZ fusion assays (figure 5*e*) with WT*/*pBAD33-*rovM* and WT*/*pBAD33 revealed the positive regulatory action of RovM on its own gene. The EMSA assay detected the dose-dependent binding of His-RovM to the *rovM* promoter–proximal fragment (figure 5*f*). Therefore, RovM has an autostimulatory effect though binding to the upstream DNA region of its own gene.

Similarly, the *rovA* deletion led to the elevated transcription of YPO1635 (electronic supplementary material, figure S15*a*–*c*) and *phoP* (electronic supplementary material, figure S16*a*–*c*), but had no effect on the transcription of *fur* (electronic supplementary material, figure S17*a*–*c*) or *rovM* (electronic supplementary material, figure S18*a*–c). His-RovA could not bind to the promoter–proximal region of YPO1635 (electronic supplementary material, figure S15*d*), *phoP* (electronic supplementary material, figure S16*d*), *fur* (electronic supplementary material, figure S17*d*) or *rovM* (electronic supplementary material, figure S18*d*)*.* Therefore, RovA indirectly represses the transcription of YPO1635*-phoPQ-*YPO1632 and *phoPQ-*YPO1632, but it does not regulate *fur* and *rovM*. The RovA concentration-dependent positive and negative regulation of its own gene has been described in our previous work [25].

### 2.5. Upregulation of *rovM* at 26°C relative to 37°C

The primer extension and real-time RT-PCR assays showed that the mRNA level of *rovM* was decreased following the shift of growth temperature from 26°C to 37°C (electronic supplementary material, figure S19*a* and S19*b*), which was further confirmed by the LacZ fusion experiments (electronic supplementary material, figure S19*c*). Thus, *rovM* transcription is upregulated at 26°C relative to 37°C.

The primer extension and LacZ fusion experiments were carried out using WT, *ΔrovM*, *C-rovM*, *ΔrovA* and *C-rovA* to characterize RovM- and RovA-dependent expression of *hmsT*; as expected, RovM and RovA promoted and repressed the transcription of *hmsT*, respectively, while the detected mRNA levels and promoter activities of *hmsT* were comparable between WT, *C-rovM* and *C-rovA* (electronic supplementary material, figure S20), confirming that the *rovM* or *rovA* mutation was non-polar.

## 3. Discussion

Data presented here (figure 1*a*) show that *Y. pestis* RovM inhibits virulence in mice, most likely through the direct repression of the transcription of *rovA*, which encodes a transcriptional activator of multiple virulence genes including the *psa* loci. Additionally, RovM promotes biofilm/c-di-GMP production by directly stimulating the transcription of *hmsT* and *hmsCDE*, and upregulates *hmsHFRS* transcription and simultaneously downregulates *hmsP* transcription, both in an indirect manner. RovM appears to be a master activator of *Y. pestis* biofilm production because it is able to modulate all four major biofilm gene loci, *hmsT*, *hmsCDE*, *hmsHFRS* and *hmsP*. By contrast, RovA inhibits *Y. pestis* biofilm formation through directly repressing *hmsT* transcription and indirectly downregulating *waaAE-coaD*, YPO1635*-phoPQ-*YPO1632 and *phoPQ-*YPO1632. *waaAE-coaD* transcription is known to be directly activated by PhoP [18]*.* Unlike its homologue in *Salmonella* [35], *Y. pestis* RovA cannot bind to the promoter region of either YPO1635*-phoPQ-*YPO1632 or *phoPQ-*YPO1632. RovA probably represses the transcription of YPO1635*-phoPQ-*YPO1632 and *phoPQ-*YPO1632 via acting on other protein or non-coding RNA regulators.

The upregulation of *rovM* accompanied by the downregulation of *rovA* has previously been reported in the flea gut [36]. As shown in this work, *rovM* expression is significantly upregulated at 26°C (flea gut temperature) relative to 37°C (warm-blooded host temperature). RovM appears to sense temperature signals to upregulate its own gene expression and in turns inhibits *rovA* expression in the flea gut. The elevated RovM production in the flea gut will promote the synthesis of attached biofilms and inhibit virulence gene expression, facilitating the establishment of a transmissible infection in fleas. Then, once the bacterium shifts to a lifestyle in the warm-blooded hosts, RovM production is decreased and RovA synthesis initiated, which in turn drives virulence factor synthesis and inhibits biofilm gene expression (figure 1*b*). Notably, cellular RovM levels were not shown to change following a temperature shift from 37°C and 26°C in *Y. pseudotuberculosis* [26], and the temperature-dependent *rovM* expression might be an example of favourable evolution to promote *Y. pestis* transmission via fleas.

It was recently reported that the deletion of *rovM* in *Y. pestis* did not affect biofilm formation and flea gut blockage [37]. Notably, the authors of this study used a low-virulence derivative of strain KIM, designated KIM6+, which lacks pCD1 [37]. This compares with the WT strain 201 in the presence of pCD1 that was used in this study. Previously, use of a pCD1-cured derivative of strain CO92 resulted in the negative regulation of biofilm formation by Hfq through promoting *hmsP* mRNA accumulation and simultaneously decreasing *hmsT* transcript stability [38]; this was confirmed using pCD1-cured strain 201 (2016, unpublished data). However, the use of WT strain 201 produced the opposite result, in which Hfq enhanced biofilm formation through positively regulating *hmsT*, *hmsCDE* and *hmsHFRS* and negatively regulating *hmsP* (2016, unpublished data). Similarly, the presence and absence of pCD1 resulted in the observation of opposite phenotypes of poly-*N*-acetylglucosamine production [39]. *Y. pestis* genetic backgrounds (e.g. presence or absence of pCD1) and even bacterial cultivation conditions would alter the relevant regulatory pathways of *Y. pestis* biofilm formation.

## 4. Material and methods

### 4.1. Bacterial strains and growth

The WT *Y. pestis* biovar *Microtus* strain 201 is avirulent to humans but highly lethal to mice [40]. Base pairs 41–362 of *rovA* (total length, 432 bp) or the entire coding region of *rovM* was replaced by the kanamycin resistance cassette using the one-step inactivation method based on the lambda phage recombination system. This generated the *Y. pestis rovA* and *rovM* null mutants (designated *ΔrovA* or *ΔrovM*, respectively). All primers used in this study are listed in the electronic supplementary material, table S1. *Y. pestis ΔhmsS* [41] was used as a reference biofilm-defective strain.

A PCR-generated DNA fragment containing the *rovA* or *rovM* coding region with its approximately 500 bp upstream promoter–proximal region and approximately 300 bp downstream transcriptional terminator region was cloned into the pACYC184 vector (GenBank accession number X06403). The recombinant plasmid was introduced into *ΔrovA* or *ΔrovM*, yielding the complemented mutant strain *C-rovA* or *C-rovM*, respectively.

A PCR-generated DNA fragment composed of the *rovM* coding region together with an upstream synthetic ribosome binding site (AGGAGGAATTCACC) was cloned between the *Xba*I and *Hind*III sites of the pBAD33 vector [42] harbouring an arabinose P_BAD_ promoter and a chloramphenicol resistance gene. Upon being verified by DNA sequencing, the recombinant plasmid pBAD33-*rovM* was introduced into WT through electrotransformation, yielding the *rovM*-overexpressed strain WT*/*pBAD33-*rovM*. The empty vector pBAD33 was also introduced into WT to generate WT/pBAD33.

### 4.2. Bacterial growth and RNA isolation

For *psaABC*-related gene regulation experiments, SBHI broth (3.7% Bacto Brain Heart Infusion (BD Biosciences), 0.5% Oxoid yeast extract, 2.5 mM CaCl_2_, 0.2% xylose, pH 6.0) was used for bacterial cultivation [43]. An overnight bacterial culture with an optical density (OD_620_) value of about 1.0 was diluted 1 : 20 into fresh SBHI for further growth at 26°C with shaking at 230 r.p.m. The cell culture with an OD_620_ of about 0.6 was transferred to 37°C, and then allowed to grow for a further 3 h prior to cell harvest.

For *rovM* autoregulation-related gene regulation and phenotypic experiments, an overnight cell culture in Luria–Bertani (LB) broth with an OD_620_ of about 1.0 was diluted 1 : 20 into fresh LB broth for further cultivation at 26°C to reach an OD_620_ of about 0.6. Then, 0.2% arabinose was added, and the culture was allowed to grow for a further 3 h.

For all other gene regulation and phenotypic assays, an overnight cell culture in LB broth with an OD_620_ of about 1.0 was diluted 1 : 20 into fresh LB broth for further cultivation at 26°C to reach an OD_620_ of about 1.0. To elicit a temperature upshift from 26°C to 37°C (for monitoring *rovM* regulation), half of the cell cultures were incubated at 37°C for 3 h while the remaining half were allowed to grow continuously at 26°C for 3 h prior to cell harvest.

Before the bacterial harvest, double-volume RNAprotect Bacteria Reagent (Qiagen) was added immediately to the cell culture. Total bacterial RNAs were extracted using TRIzol Reagent (Invitrogen) [15,16]. The RNA quality was monitored by agarose gel electrophoresis, and its quantity was determined by spectrophotometry.

### 4.3. Primer extension assay

For the primer extension assay [15,16], an oligonucleotide primer complementary to a portion of the RNA transcript of each indicated gene was used to synthesize cDNAs from RNA templates. About 10 µg of total RNA from each strain was annealed with 1 pmol of [*γ*-^32^P] end-labelled reverse primer using a Primer Extension System (Promega) according to the manufacturer's instructions. The same labelled primer was also used for sequencing with the fmol^®^ DNA Cycle Sequencing System (Promega). The primer extension products and sequencing materials were concentrated and analysed in a 6% polyacrylamide/8 M urea gel. The result was detected by autoradiography (Kodak film).

### 4.4. Quantitative RT-PCR

Gene-specific primers were designed to produce amplicons for target genes. Contaminating DNA in the RNA samples was removed using the Ambion DNA-free™ Kit (Applied Biosystems). cDNAs were generated by using 5 µg of RNA and 3 µg of random hexamer primers. Real-time PCR was performed using the LightCycler system (Roche) and the SYBR Green master mix [44,45]. Based on the standard curves of 16S rRNA expression, the relative mRNA level was determined by calculating the threshold cycle (ΔCt) of target genes via the classic ΔCt method. Negative controls used cDNA generated without reverse transcriptase as templates. Reactions containing primer pairs without template were also included as blank controls. The 16S rRNA gene was used as an internal control for normalization.

### 4.5. Lacz reporter fusion and β-galactosidase assay

The promoter–proximal DNA region of each gene tested was prepared by PCR with Takara ExTaq DNA polymerase using *Y. pestis* 201 genomic DNA as template. This was then cloned directionally into the *Hind*III*–Bam*HI site of the transcriptional fusion vector pRW50 [46] that contained a promotorless *lacZ* reporter gene. The clone was verified by DNA sequencing. Each *Y. pestis* strain tested was transformed with the recombinant plasmids, and an empty plasmid was introduced into each strain as a negative control. β-Galactosidase activity was measured in extracts from cells cultivated as above using the β-galactosidase enzyme assay system (Promega) [15,16].

### 4.6. Preparation of 6× His-tagged RovA (His-RovA) and RovM (His-RovM) protein

The preparation of purified RovA [24] or RovM [26] protein was performed as previously described. The entire coding region of *rovA* or *rovM* was amplified from *Y. pestis* 201 or EV76, respectively, and cloned directionally into the *Bam*HI and *Hind*III or *Bam*HI and *Sal*I sites of plasmid pET28a (Novagen), respectively. The recombinant plasmids were transformed into *E. coli* BL21 (DE3) cells (Novagen). Expression of His-RovA or His-RovM protein was induced by the addition of 1 mM or 2 mM isopropyl-beta-d-thiogalactoside, respectively. His-RovA or His-RovM were purified under native conditions using QIAexpressionist™ Ni–NTA affinity chromatography (Qiagen). The purified, eluted protein was concentrated with the Amicon Ultra-15 (Millipore) to a final concentration of 0.5–0.7 mg ml^−1^ in the storage buffer containing phosphate-buffered saline (PBS, pH 8.0) plus 20% glycerol. The protein purity was verified by sodium dodecyl sulfate polyacrylamide gel electrophoresis with silver staining.

### 4.7. Electrophoretic mobility shift assay

For EMSA [15,16], promoter–proximal DNA regions were prepared by PCR amplification. EMSA was performed using the Gel Shift Assay Systems (Promega). The 5′ ends of DNA were labelled using [*γ*-^32^P] ATP and T4 polynucleotide kinase. DNA binding was performed in a 10 µl volume containing binding buffer (100 µM MnCl_2_, 1 mM MgCl_2_, 0.5 mM DTT, 50 mM KCl, 10 mM Tris–HCl (pH 7.5), 0.05 mg ml^−1^ sheared salmon sperm DNA, 0.05 mg ml^−1^ BSA and 4% glycerol), labelled DNA (1000–2000 c.p.m µl^−1^) and increasing amounts of His-RovA or His-RovM. We included two control reactions: one contained the specific DNA competitor (unlabelled promoter DNA regions; cold probe), whereas the other was the non-specific protein competitor (rabbit anti-F1-protein polyclonal IgG antibody). After incubation at room temperature for 30 min, the products were loaded onto a native 4% (w/v) polyacrylamide gel and electrophoresed in 0.5× Tris–borate buffer containing 100 µM MnCl_2_ for 30 min at 220 V. Radioactive species were detected by autoradiography.

### 4.8. Biofilm-related assays

Four different methods [47,48] of biofilm-related assays were used (i) crystal violet staining of the *in vitro* biofilm masses attached to the well walls when bacteria were grown in polystyrene microtitre plates; (ii) determination of the percentages of fourth-stage larvae and adults (L4/adult) of *Caenorhabditis elegans* after the incubation of nematode eggs on *Y. pestis* lawns, which negatively reflected the bacterial ability to produce biofilms; (iii) observation of the rugose colony morphology of bacteria grown on LB agar plates, which positively reflected the bacterial ability to synthesize biofilm matrix exopolysaccharide; and (iv) determination of intracellular c-di-GMP levels by a chromatography-coupled tandem mass spectrometry method.

### 4.9. Murine infection model

All animal experiments were conducted in accordance with Guidelines for Welfare and Ethics of Laboratory Animals of China. Bacterial cultures were washed twice with PBS (pH 7.2), and then subjected to serial 10-fold dilutions with PBS. Appropriate dilutions were plated onto He's agar plates (LandBridge) to calculate the numbers of colony-forming units (CFU). For each strain tested, 0.1 ml of the 10^3^ CFU ml^−1^ bacterial suspension was inoculated by s.c. injection at the inguinal region or by i.v. injection via the vena caudalis into each of 10 female BALB/c mice (aged six to eight weeks old). The numbers of mice that died at specified times were then calculated and used to draw a survival curve with GraphPad Prism v. 5.0. *p-*Values were calculated with the log-rank (Mantel–Cox) test and the Gehan–Breslow–Wilcoxon test; *p* < 0.01 was considered to indicate statistical significance.

### 4.10. Experimental replicates and statistical methods

For real-time RT-PCR, LacZ fusion, crystal violet staining of biofilms, and the determination of L4/adult nematodes or c-di-GMP, experiments were performed with at least three independent bacterial cultures/lawns, and values were expressed as mean ± standard deviation. The paired Student's *t*-test was performed to determine significant differences; *p* < 0.01 was considered to indicate statistical significance. For primer extension, EMSA and colony morphology observation, representative data from at least two independent biological replicates are shown.

## Supplementary Material

Supplementary figures and tables
