# Supplementary material for: Reciprocal regulation of Yersinia pestis biofilm formation and virulence by RovM and RovA
Source: Open Biol. 2016 Mar 16;6(3):150198. doi: 10.1098/rsob.150198 (PMC4821237; doi:10.1098/rsob.150198)
Supplement: Supplementary figures and tables [file rsob150198supp1.docx]

**
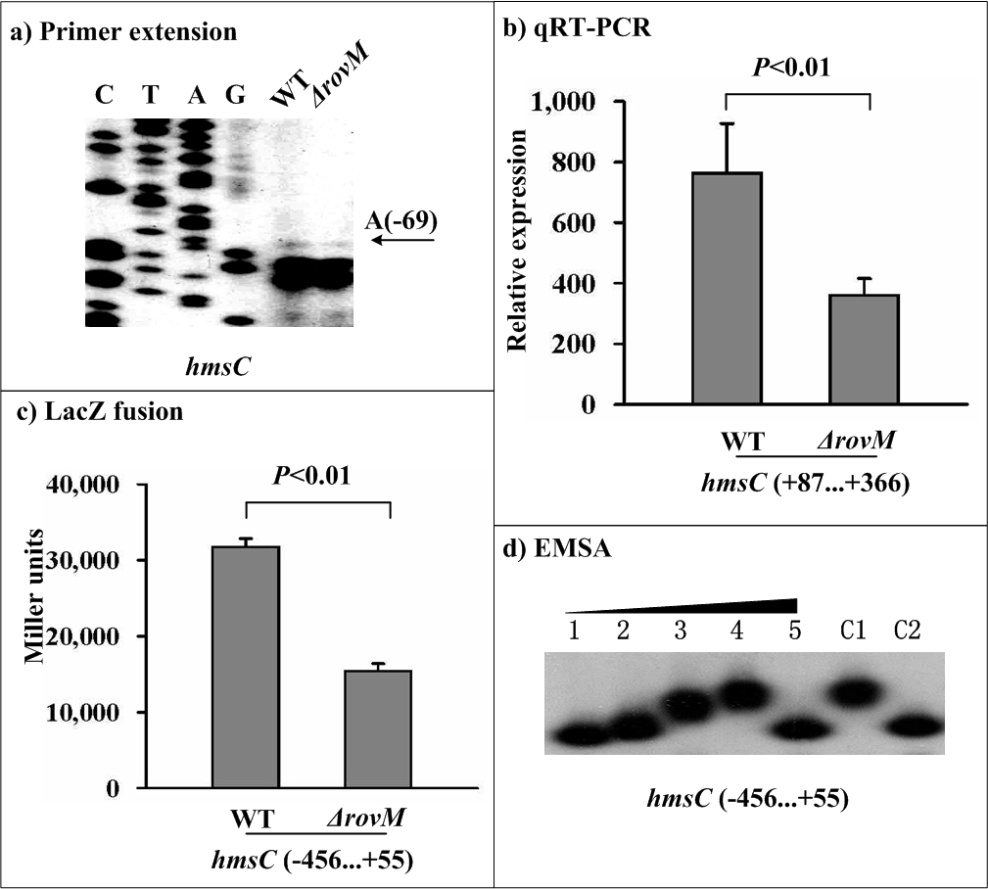
**

**Figure S1. Direct activation of *hmsC* by RovM.** See Figure 3 for the annotations of primer extension (**a**), quantitative RT-PCR (**b**), LacZ fusion (**c**), and EMSA (**d**).

**
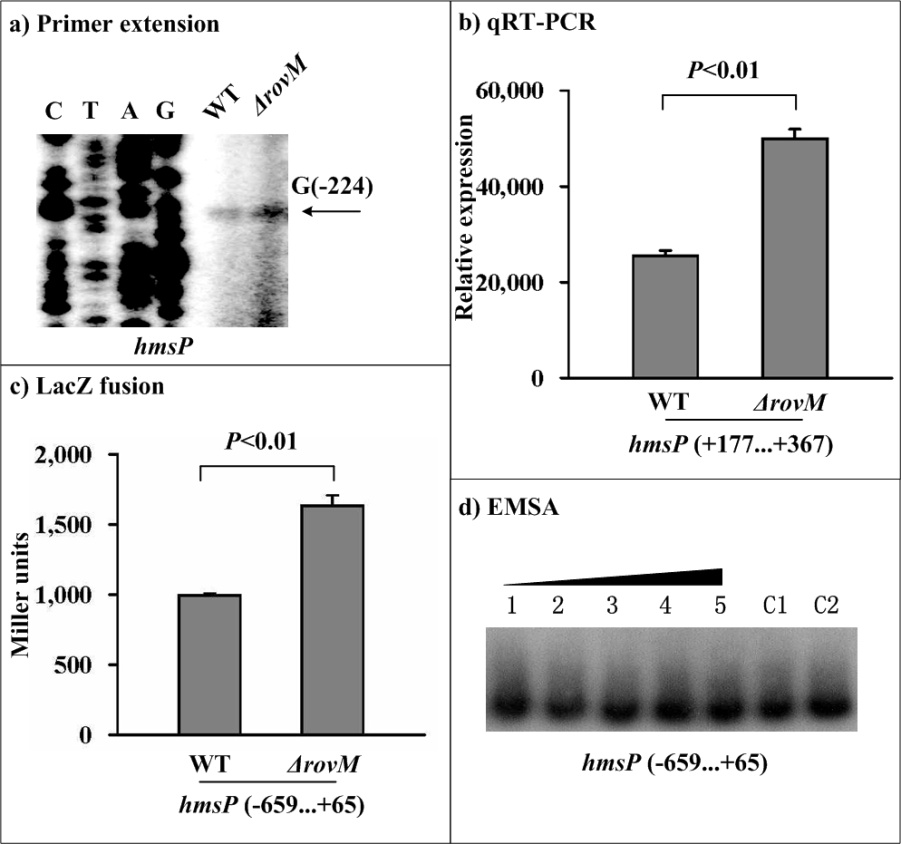
**

**Figure S2. Indirect repression of *hmsP* by RovM.** See Figure 3 for the annotations of primer extension (**a**), quantitative RT-PCR (**b**), LacZ fusion (**c**), and EMSA (**d**).


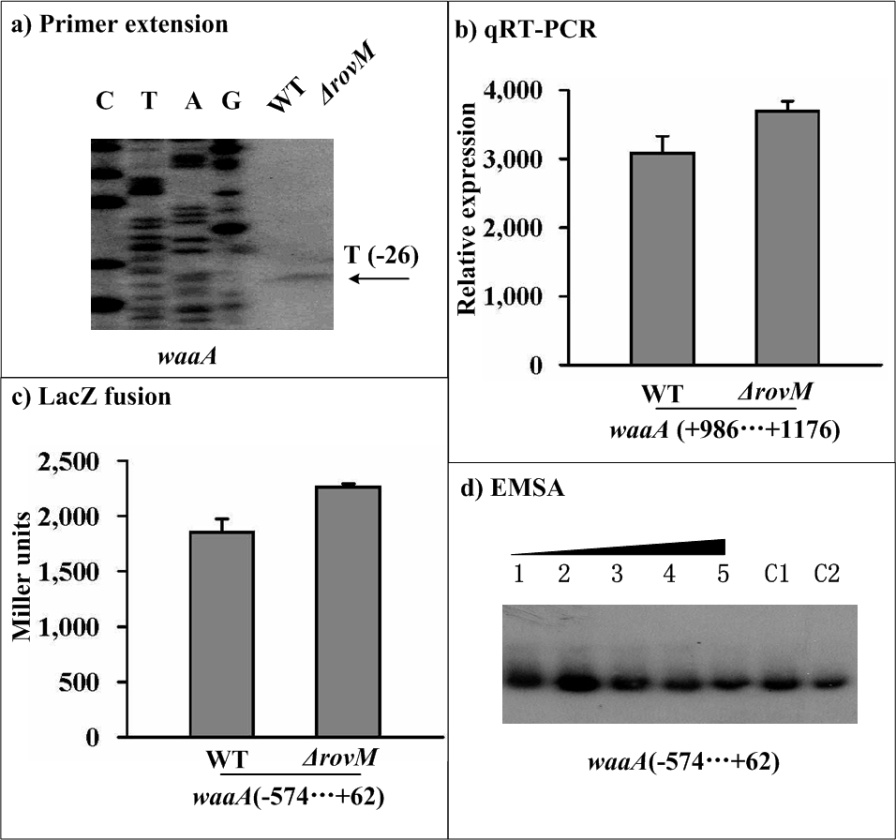


**Figure S3. RovM had no regulatory effect on *waaA* transcription.** See Figure 3 for the annotations of primer extension (**a**), quantitative RT-PCR (**b**), LacZ fusion (**c**), and EMSA (**d**).


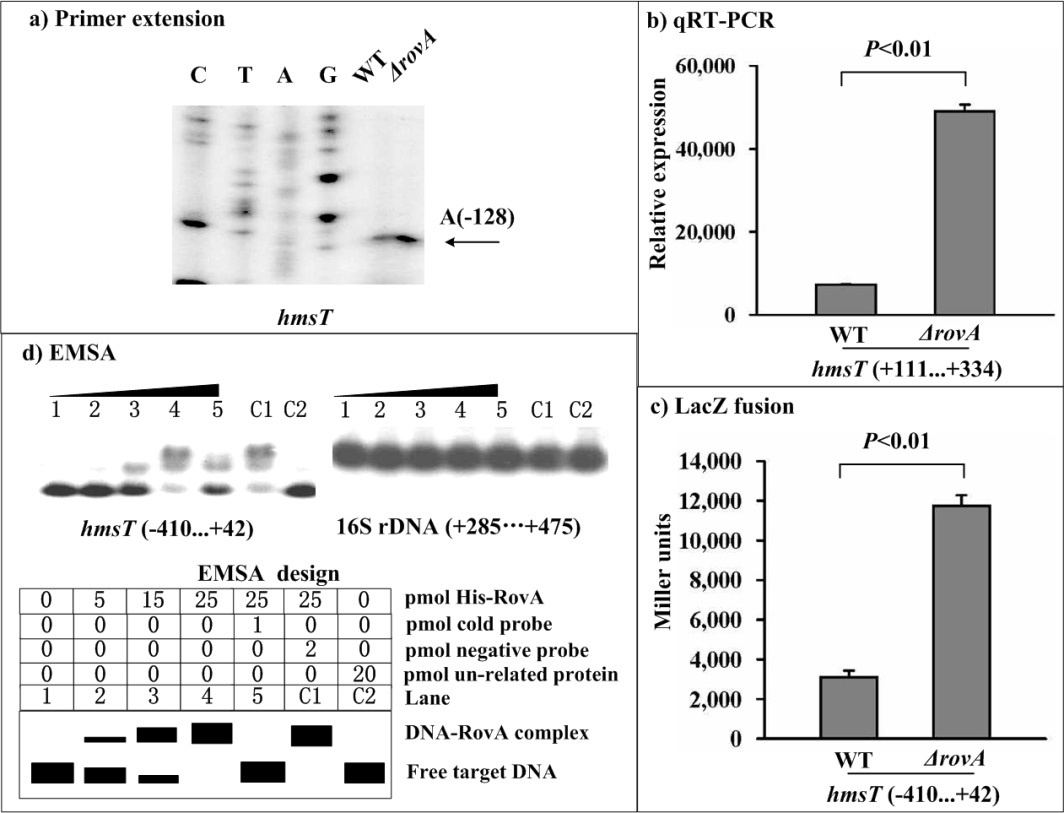


**Figure S4. Direct repression of *hmsT* by RovA.** See Figure 3 for the annotations of primer extension (**a**), quantitative RT-PCR (**b**), LacZ fusion (**c**), and EMSA (**d**).


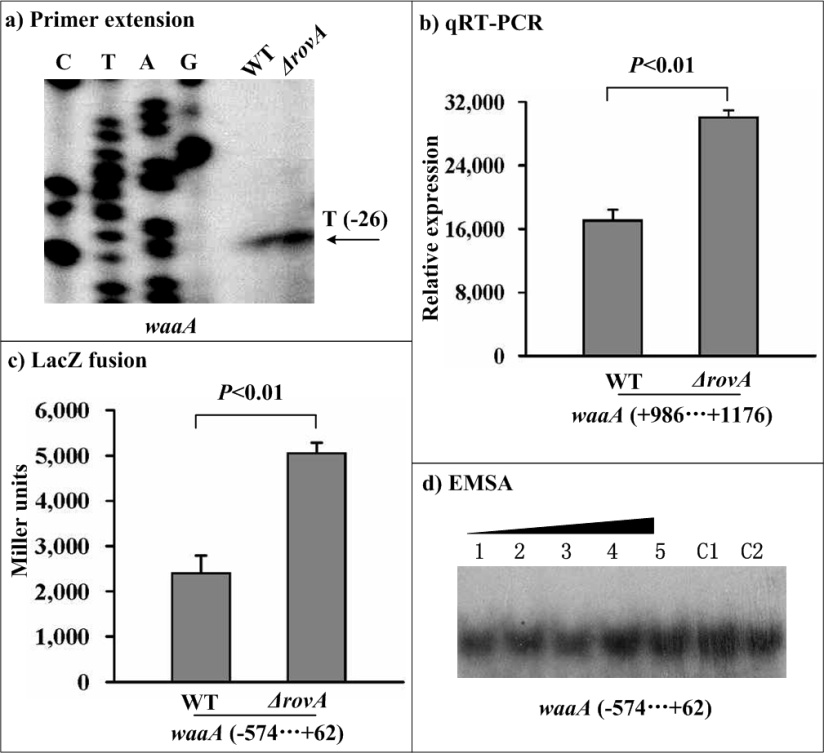


**Figure S5. Indirect repression of *waaA* by RovA.** See Figure 3 for the annotations of primer extension (**a**), quantitative RT-PCR (**b**), LacZ fusion (**c**), and EMSA (**d**).


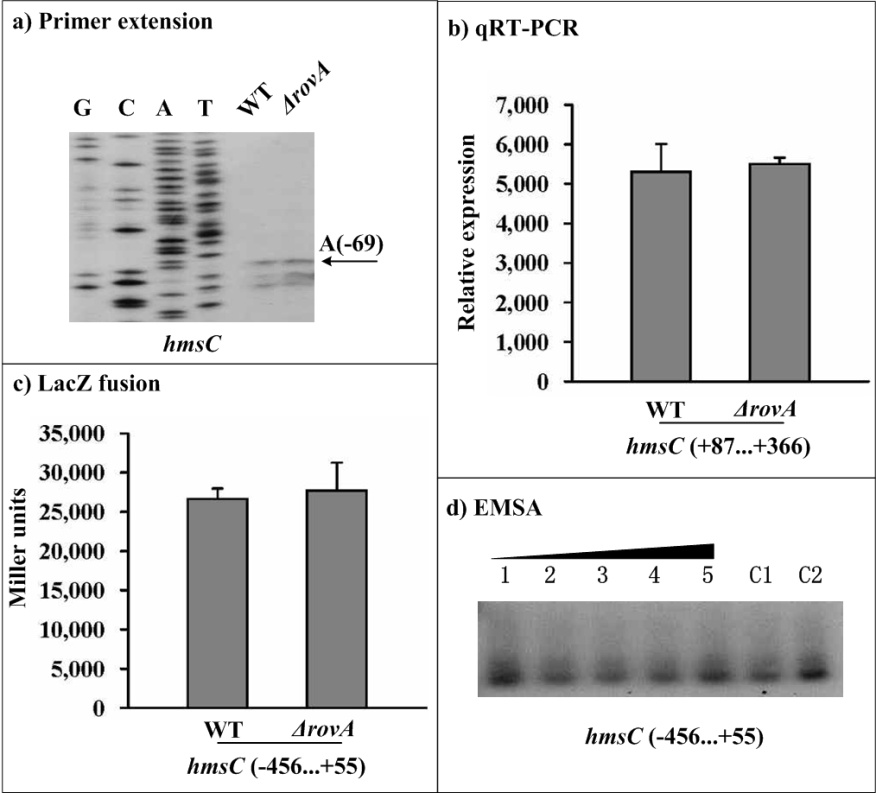


**Figure S6. RovA had no regulatory effect on *hmsC* transcription.** See Figure 3 for the annotations of primer extension (**a**), quantitative RT-PCR (**b**), LacZ fusion (**c**), and EMSA (**d**).


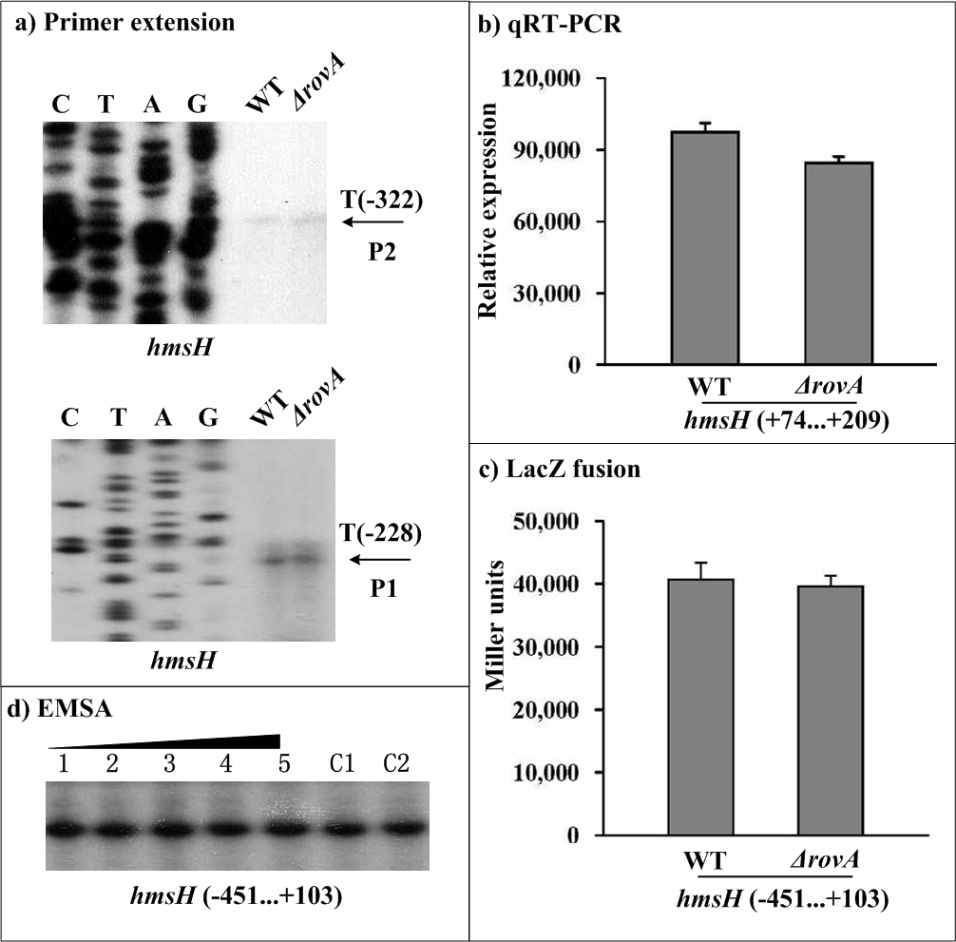


**Figure S7. RovA had no regulatory effect on *hmsH* transcription.** See Figure 3 for the annotations of primer extension (**a**), quantitative RT-PCR (**b**), LacZ fusion (**c**), and EMSA (**d**).

**
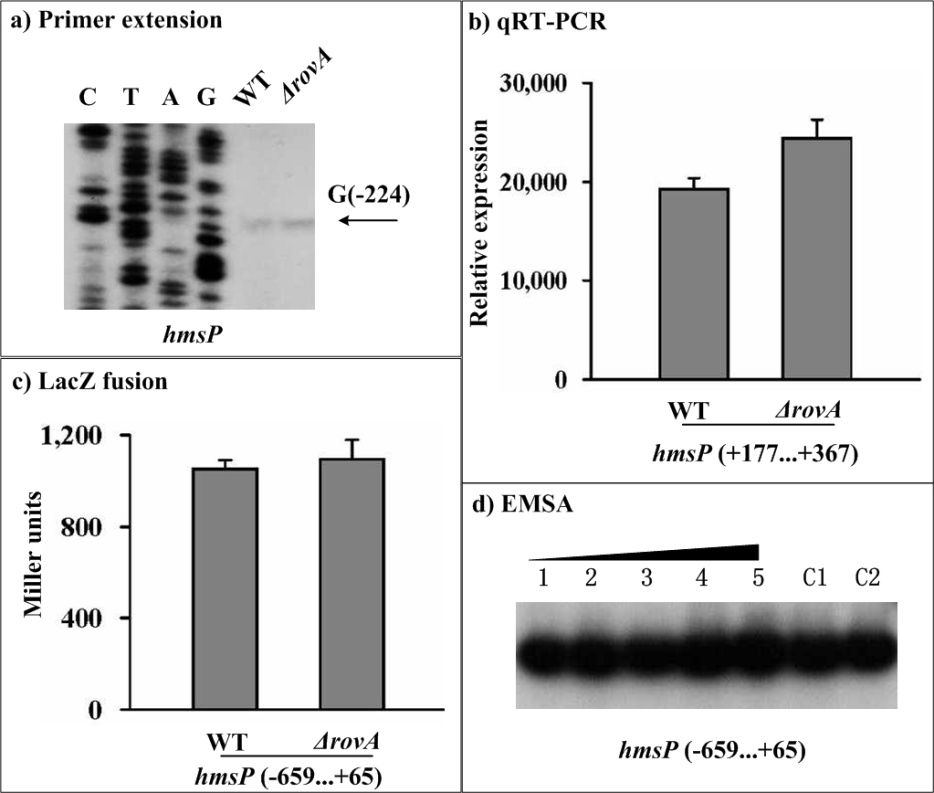
**

**Figure S8. RovA had no regulatory effect on *hmsP* transcription.** See Figure 3 for the annotations of primer extension (**a**), quantitative RT-PCR (**b**), LacZ fusion (**c**), and EMSA (**d**).


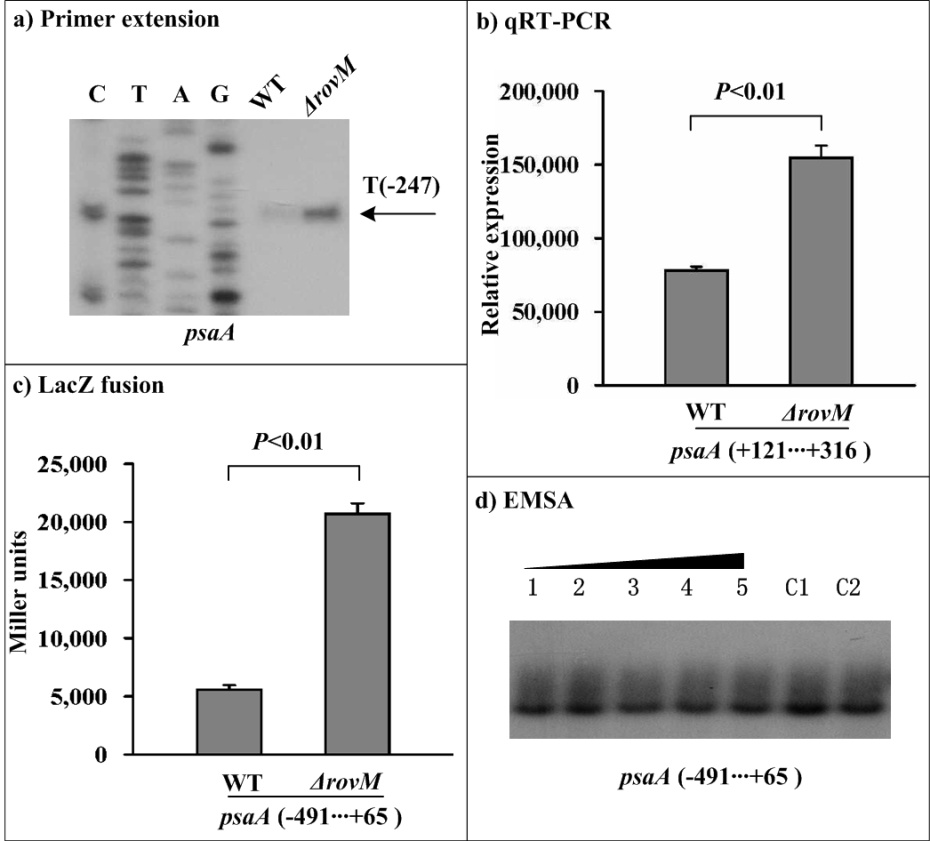


**Figure S9. Indirect repression of *psaA* by RovM.** See Figure 3 for the annotations of primer extension (**a**), quantitative RT-PCR (**b**), LacZ fusion (**c**), and EMSA (**d**).


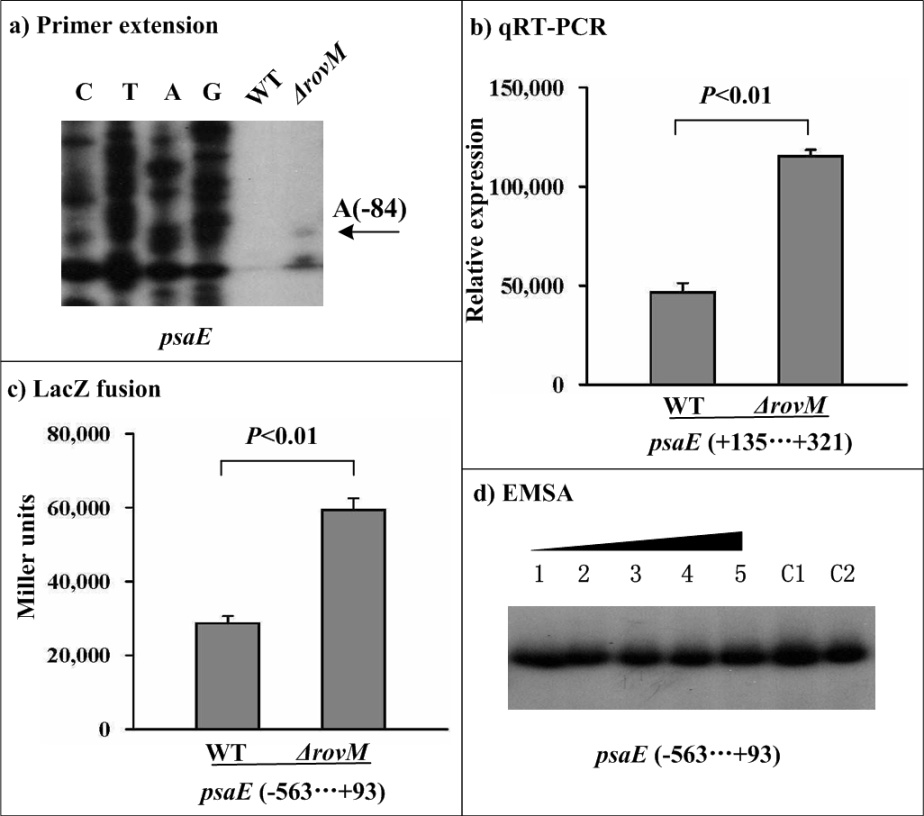


**Figure S10. Indirect repression of *psaE* by RovM.** See Figure 3 for the annotations of primer extension (**a**), quantitative RT-PCR (**b**), LacZ fusion (**c**), and EMSA (**d**).


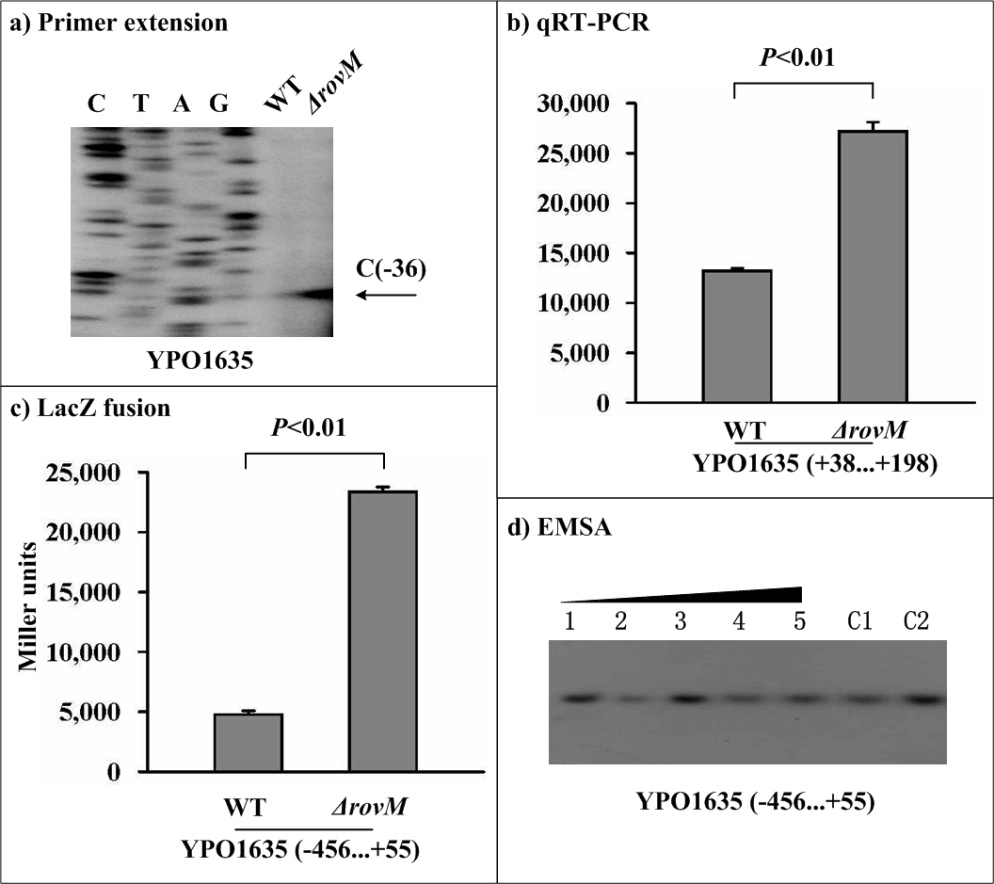


**Figure S11. Indirect repression of YPO1635 by RovM.** See Figure 3 for the annotations of primer extension (**a**), quantitative RT-PCR (**b**), LacZ fusion (**c**), and EMSA (**d**).


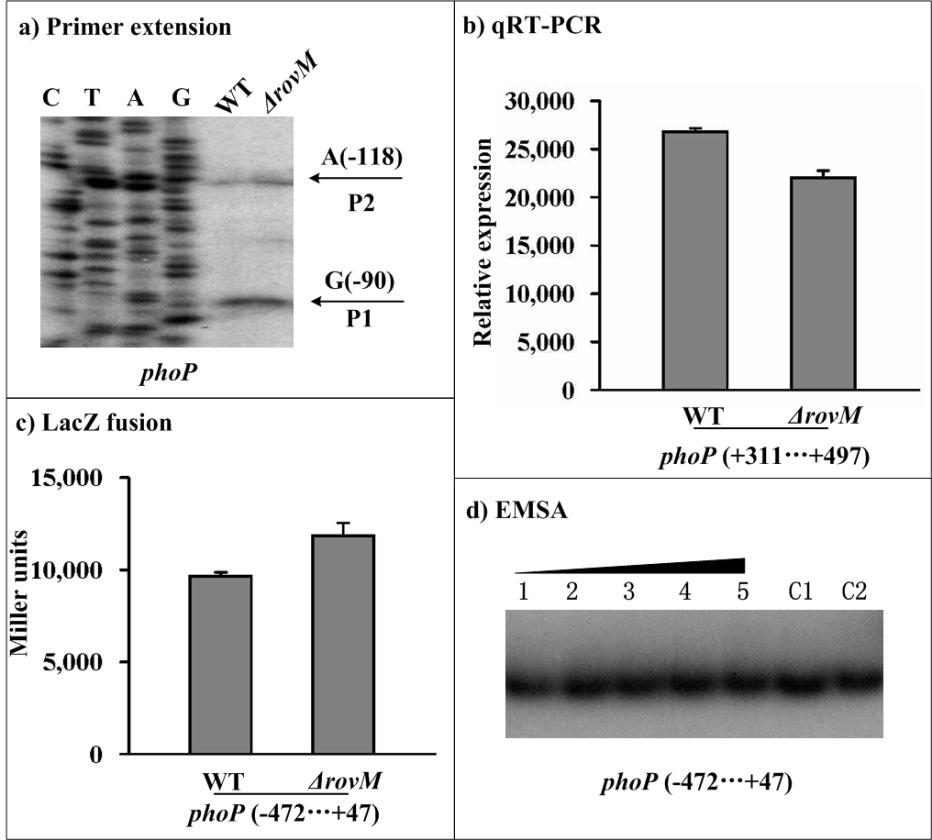


**Figure S12. RovM had no regulatory effect on *phoP* transcription.** See Figure 3 for the annotations of primer extension (**a**), quantitative RT-PCR (**b**), LacZ fusion (**c**), and EMSA (**d**).


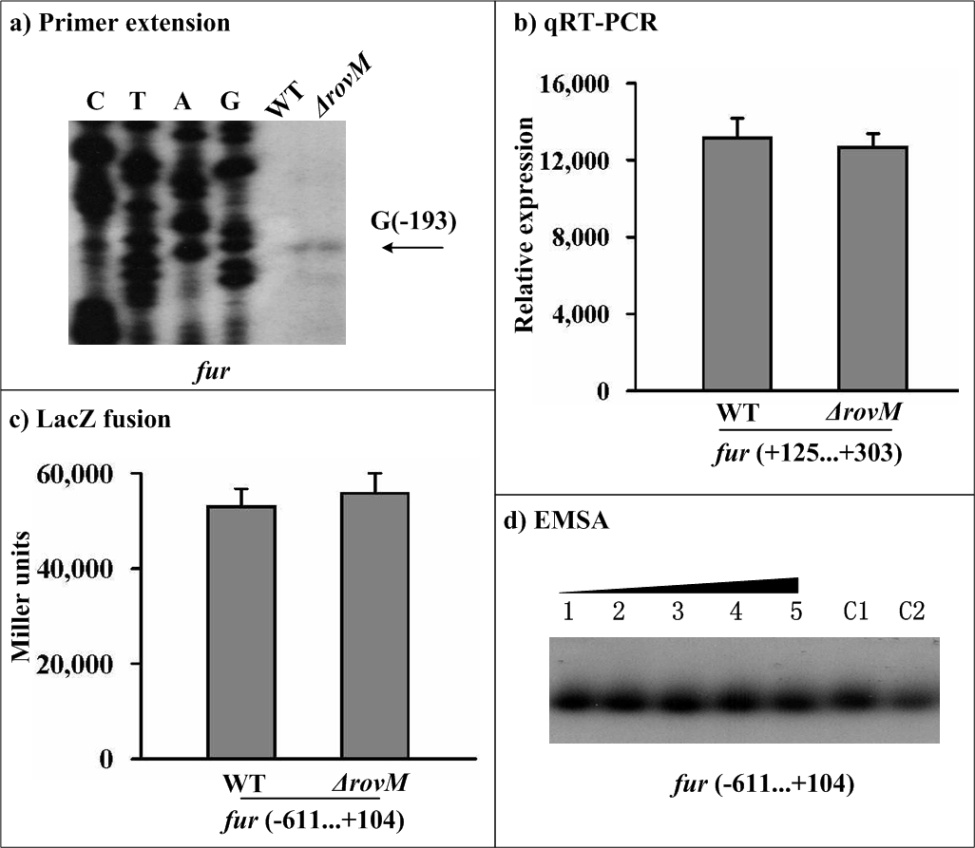


**Figure S13. RovM had no regulatory effect on *fur* transcription.** See Figure 3 for the annotations of primer extension (**a**), quantitative RT-PCR (**b**), LacZ fusion (**c**), and EMSA (**d**).


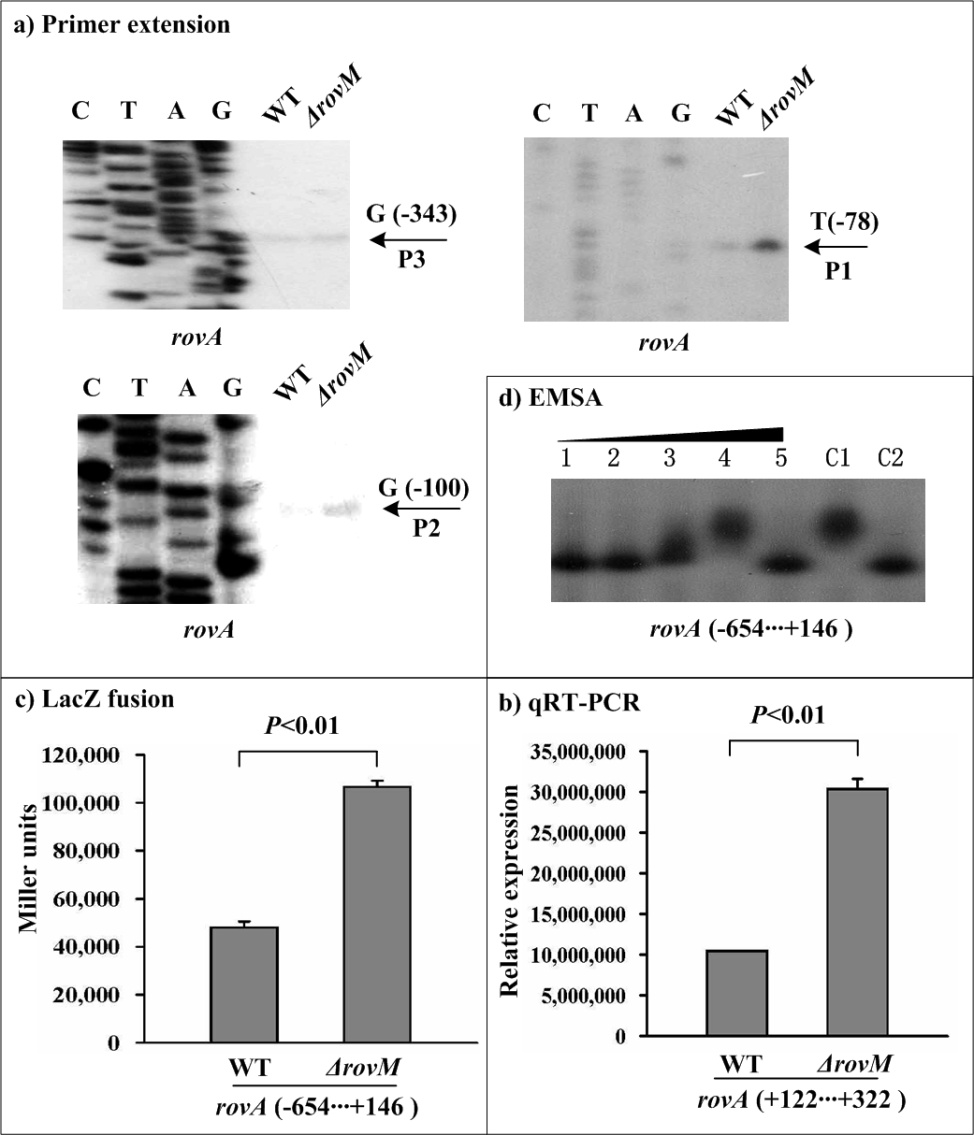


**Figure S14. Direct repression of *rovA* by RovM.** See Figure 3 for the annotations of primer extension (**a**), quantitative RT-PCR (**b**), LacZ fusion (**c**), and EMSA (**d**).


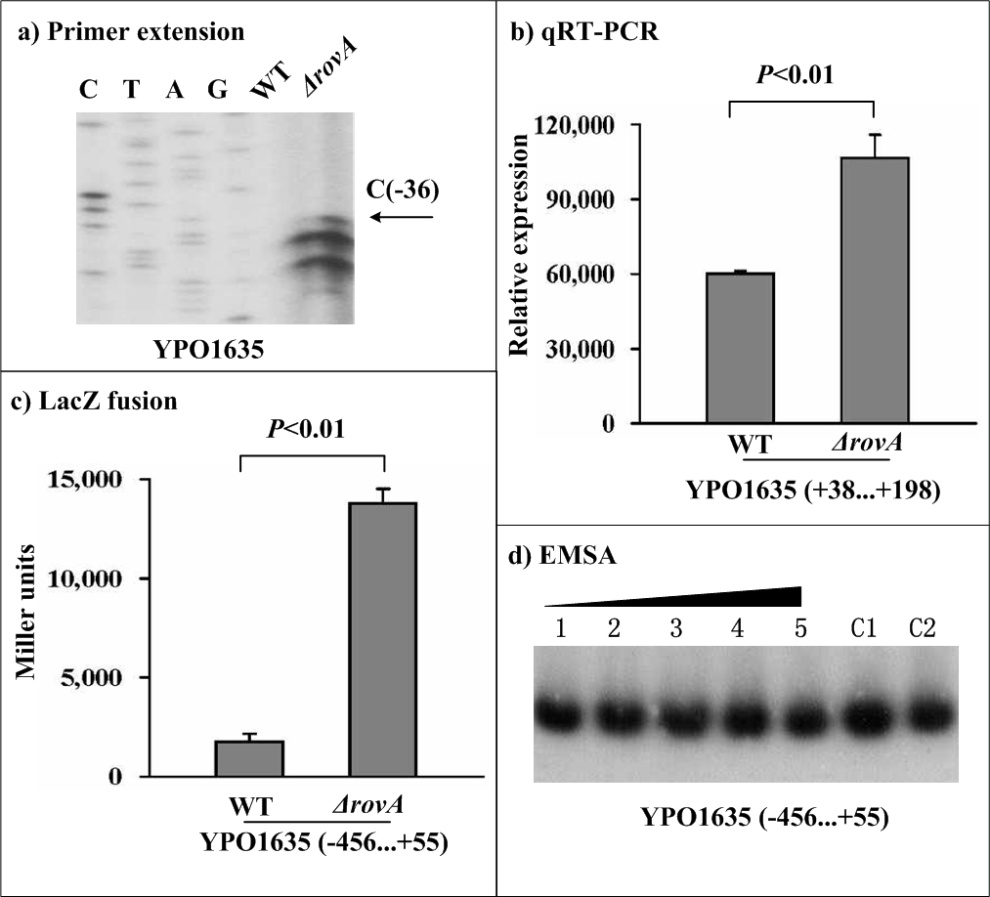


**Figure S15. Indirect repression of YPO1635 by RovA.** See Figure 3 for the annotations of primer extension (**a**), quantitative RT-PCR (**b**), LacZ fusion (**c**), and EMSA (**d**).

**
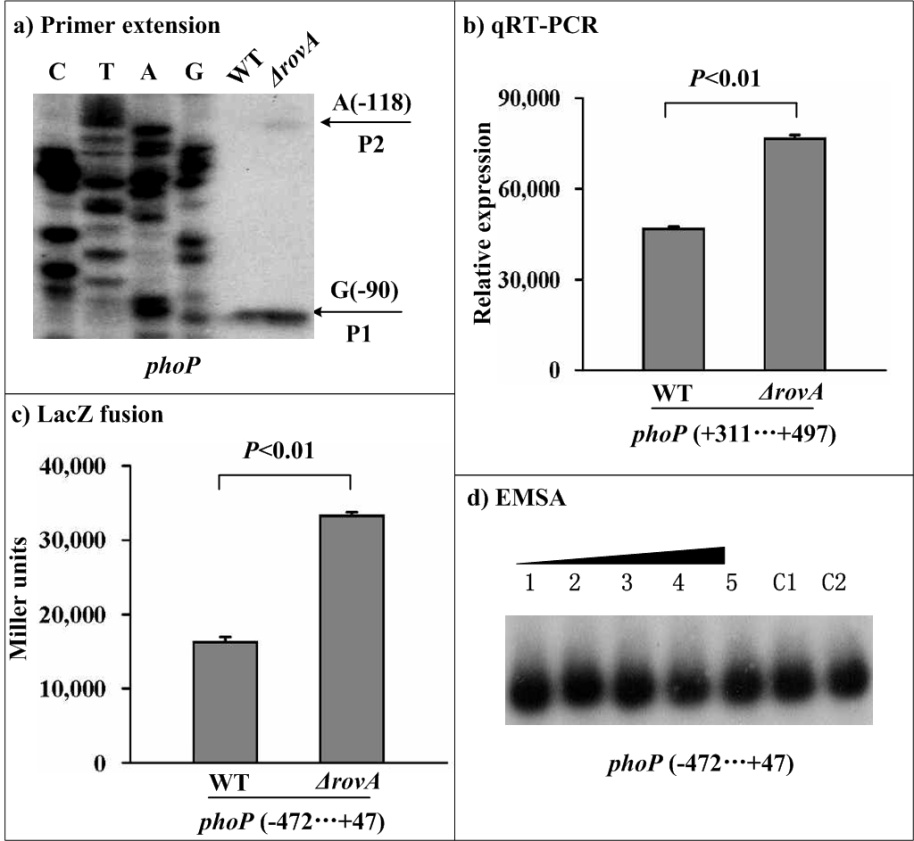
**

**Figure S16. Indirect repression of *phoP* by RovA.** See Figure 3 for the annotations of primer extension (**a**), quantitative RT-PCR (**b**), LacZ fusion (**c**), and EMSA (**d**).


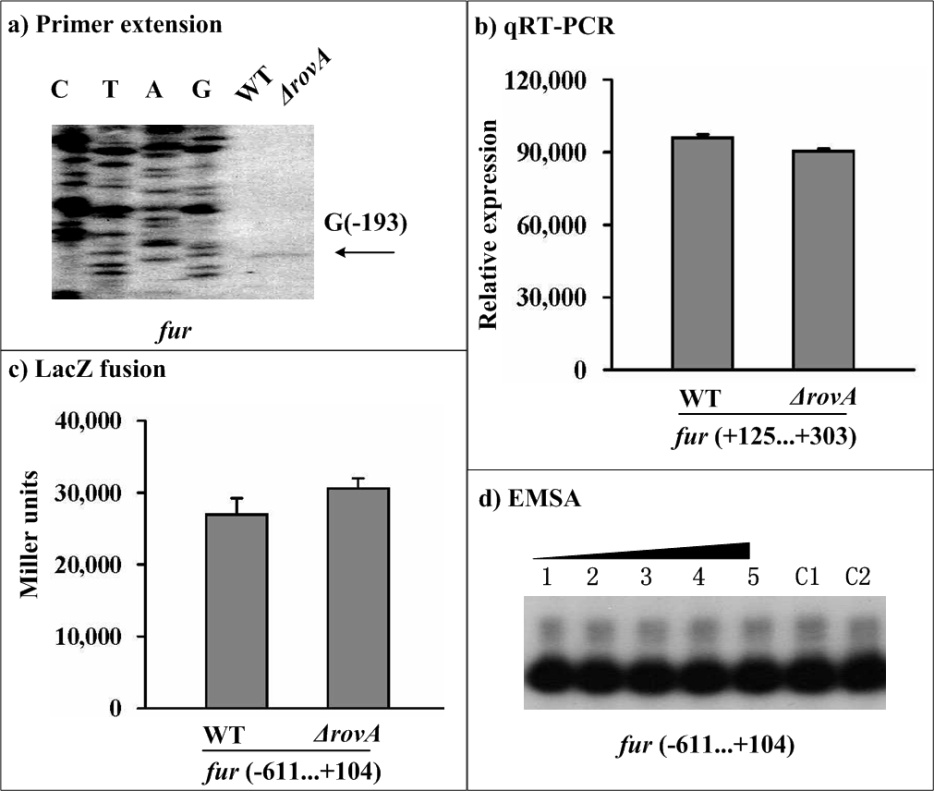


**Figure S17. RovA had no regulatory effect on *fur* transcription.** See Figure 3 for the annotations of primer extension (**a**), quantitative RT-PCR (**b**), LacZ fusion (**c**), and EMSA (**d**).


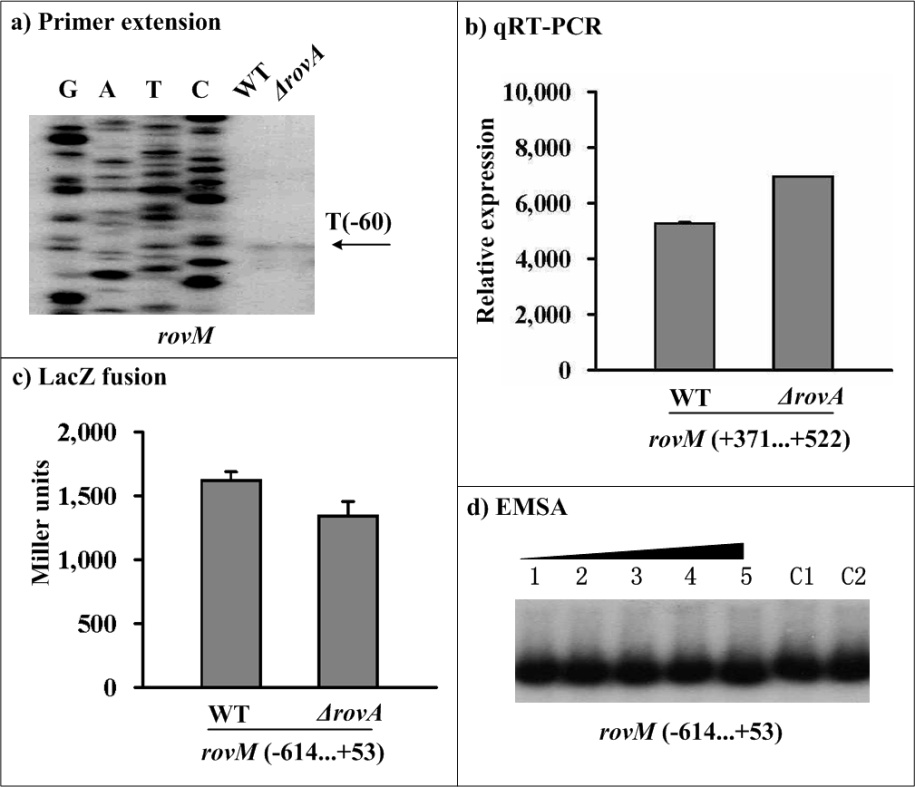


**Figure S18. RovA had no regulatory effect on *rovM* transcription.** See Figure 3 for the annotations of primer extension (**a**), quantitative RT-PCR (**b**), LacZ fusion (**c**), and EMSA (**d**).


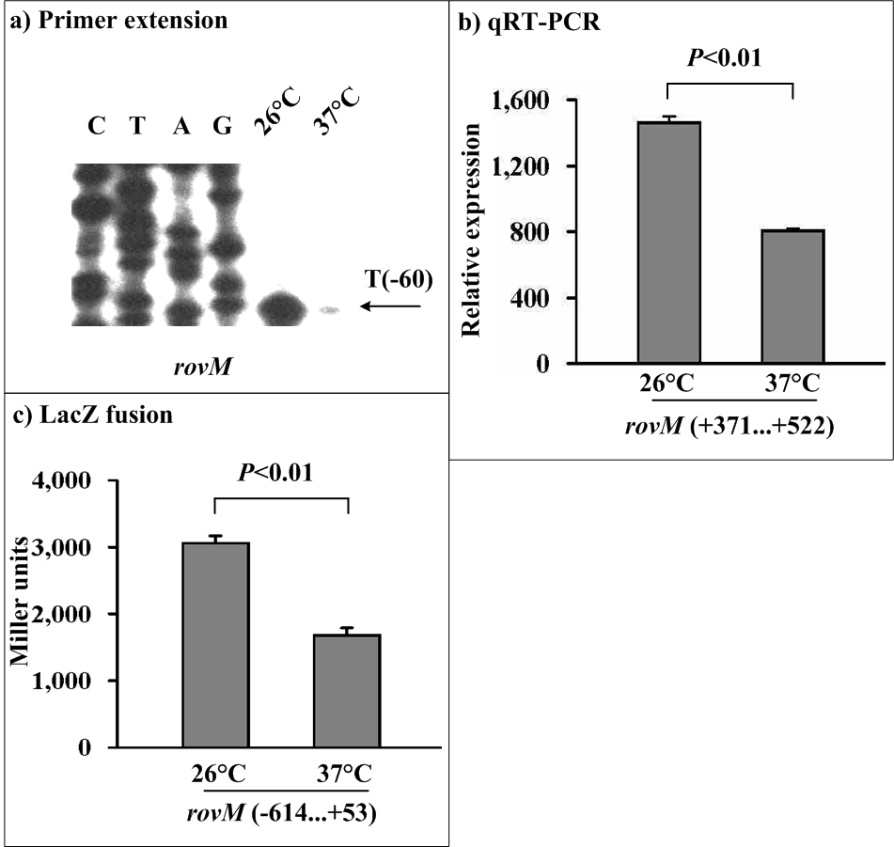


**Figure S19. Down-regulation *of rovM* upon temperature upshift from 26 to 37°C.** See Figure 3 for the annotations of primer extension (**a**), quantitative RT-PCR (**b**), and LacZ fusion (**c**).


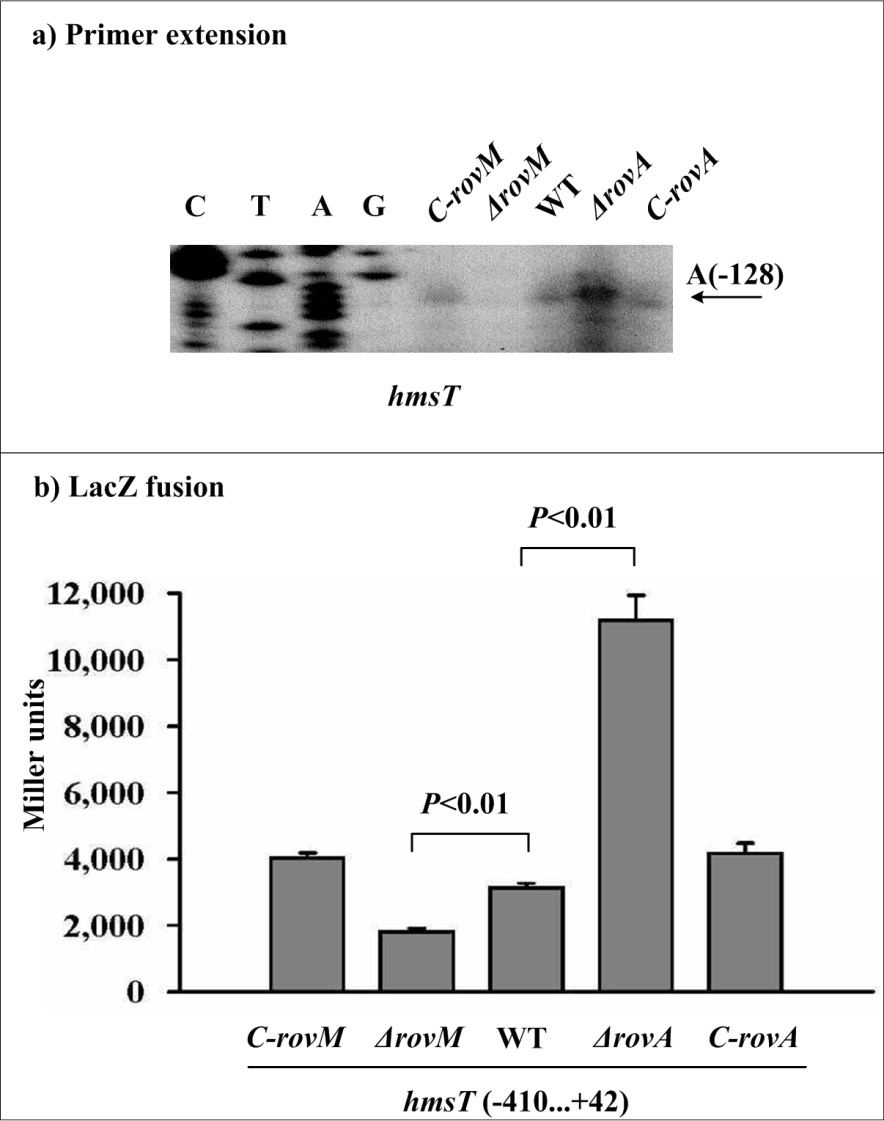


**Figure S20. Primer extension and LacZ fusion assays for validation of non-polar mutation of *rovM* or *rovA*.** The *rovM* or *rovA* null mutant (*ΔrovM* or *ΔrovA*, respectively) was generated from the wild-type strain (WT), and then the corresponding complemented mutant strain (*C-rovM* or *C-rovA*, respectively) was constructed. Herein, the primer extension and LacZ fusion experiments were carried out using the above five strains. See Figure 3 for the annotations of primer extension (**a**) and LacZ fusion (**b**). RovM and RovA promoted and repressed the transcription of *hmsT*, respectively, while the detected mRNA levels and promoter activities of *hmsT* were comparable between WT, *C-rovM* and *C-rovA*, which confirming that the *rovM* or *rovA* mutation was nonpolar.

**Table S1. Primers used in this study**

| **Target gene** | **Primers (forward/reverse, 5′-3′)** |
| --- | --- |
| **Gene mutation** | |
| *rovM* | ATGACAAATGCAAATCGTCCGATAATTAATCTCGACCTCGAGATTGCAGCATTACACG/TTAATCTTCATCACCTGTCAAATAATCGGAATCCAATATTTGTAACGCACTGAGAAGC |
| *rovA* | TTGGAATCGACATTAGGATCTGATCTAGCACGATTAGTTCAGATTGCAGCATTACACG/CTCAAGCTTATCGATTAGGCCTGATAACACTGCAATTTCATGTAACGCACTGAGAAGC |
| *hmsS* | CGATACCGTTGAGGATTTATATTCTCAGCGGTTTGACGACAGATTGCAGCATTACACG/TATCCAGAACTTTTACGGATTCATAATAGAGTGGCGCGCTTGTAACGCACTGAGAAGC |
| **Gene complementation** | |
| *rovM* | TCTGGATCCGGCATTAAACCGCATCGTATC/GCGGTCGACATCCTGTTCTTCCACTCAGTAG |
| *rovA* | GATCGATATCGCTCAGTTGCCGCCTTC/GATCGGATCCCTGCTGTGAATAAAGTCTTTGAAC |
| **Protein overexpression** | |
| *rovM* | TCTGGTACCAGGAGGAATTCACCatgacaaatgcaaatcgtccg/GCGCTGCAGTTAATCTTCATCACCTGTCA |
| **Protein expression** | |
| *rovM* | TCTGGATCCATGACAAATGCAAATCGTCCG/GCGGTCGACTTAATCTTCATCACCTGTCA |
| *rovA* | GCGGGATCCTTGGAATCGACATTAGGATC/GCGGTCGACTTACTTAGTTTGTAATTGAATA |
| **Primer extension** | |
| *hmsT* | /GGTATTTATTCCGACATCACGAC |
| *hmsC* | /AGTAGCGGTAGTCATTTTTACG |
| *hmsH* | / TATTGTTGCAAAGTCATTATAGGAT |
| *hmsP* | /CCATCGAGTAAGTTGTGATCC |
| *waaA* | /GTAAACGCAGCCAAATCAGAG |
| *psaA* | /TAACTCAGTCGCAGACCTATAG |
| *psaE* | /TACTGTCACCAATTATTA |
| YPO1635 | /CAGGGCAAGGGATAATAGGG |
| *phoP* | /ACCCGCATACACCAATCCTT |
| *fur* | /CCAAATGAAAACGGTGGTTG |
| *rovA* | /GTATCCTCATTACCCAGCATCG  /GTGCTAGATCAGATCCTAATGTCG |
| *rovM* | /GTTCTAAGCAGATCGAGGTCG |
| **LacZ reporter fusion** | |
| *hmsT* | GCGGAATTCGCCCAGTACAGGTAACAAGG/GCGGGATCCCTGATCGTAGGAGTGGCTATTC |
| *hmsC* | TCTGGATCCCTTACTGGTTGCTATTGCC/TCTAAGCTTGAGGTTCATGATGTTCATCA |
| *hmsH* | GCGGGATCCACTTTGCTGAAGACTTGTCACG/GCGAAGCTTCCGCCATAGCAGGATTAACG |
| *hmsP* | GCGGGATCCAGCGATGGTAGAAGTGAATCAG/GCGAAGCTTTTGCGATACTCTAATGGAAGGC |
| *waaA* | GCGGGATCCTGGATCGCCCAAACATTACG/GCGAAGCTTAAACGCAGCCAAATCAGAGG |
| *psaA* | GCGGGATCCGGCGTCTGTCTATATTGGTATC/GCGAAGCTTCCACAAGCAGCGATCATTAG |
| *psaE* | GCGGGATCCGTGATCCGATGCGTGTCTTG/GCGAAGCTTTAACAGCAGCACCTCATTCTTG |
| YPO1635 | GCGGGATCCCCGACTCGACCGTGCTAC/GCGAAGCTTATGGCGACACTACAGGAACC |
| *phoP* | CCGGAATTCTGATGCCAGCAAAGACG/CGCGGATCCAGATGGTGACGCAACAAC |
| *fur* | TCTGGATCCCTGAGTATTTCTGTGATGCGATG/TCTAAGCTTCTGACGTGGTGACACGCAGG |
| *rovA* | GCGGGATCCCGTTCGTTACTCTGCCCATC/GCGAAGCTTTTGTGATTGCTCTGGTGGTAAAC |
| *rovM* | TCTGGATCCAAAGAGAACATTCAGCAGCCG/TCTAAGCTTGTTCTAAGCAGATCGAGGTCG |
| ***Quantitative RT-PCR*** | |
| *hmsT* | CAGTATGCTATCATCGTCGC/GTAGACCGATGAGGATTG |
| *hmsC* | GGGCGTTTATCTATTCTTAC/GAGTGAGTTATTGGGAAGTG |
| *hmsH* | CTGGCTTTGTCGTTAATCCTG/TGCCCACTCTGCAATGGAC |
| *hmsP* | GGTAAGGCGCTCATTAACGA/TATCAACGCTGAGTATGGCC |
| *waaA* | TTGAACGTGGCGGTCATAAC/GAGGCGGCAATCTTCATCAG |
| *psaA* | GTCAAGCAGGGAAACACATTC/AACCAACATAGTCACCATCGG |
| *psaE* | TGAATTACTGACAACCTGTTGG/ TTCGGTGCTGCCATCATC |
| YPO1635 | GTTCCTGTAGTGTCGCCATG/ACCGCCGTTAAGATAAATCCC |
| *phoP* | ATCTGGAAGAGGTCATTGC/CTGCGTTGCGGATAAGG |
| *fur* | TACTGATCGATATTGGTG/ CTCGATCACTTTGCCGCA |
| *rovA* | ATCGTTTACCACCAGAGCAATC/AATCACGCCATCAACCTGTTC |
| *rovM* | GCGATAGATGTGCGGGTAAAG/GCTCATCCATCACCACTAAAGG |
| **EMSA** | |
| *hmsT* | GCCCAGTACAGGTAACAAGG/CTGATCGTAGGAGTGGCTATTC |
| *hmsC* | CTTACTGGTTGCTATTGCC/GAGGTTCATGATGTTCATCA |
| *hmsH* | ACTTTGCTGAAGACTTGTCACG/ CCGCCATAGCAGGATTAACG |
| *hmsP* | GCGGGATCCAGCGATGGTAGAAGTGAATCAG/GCGAAGCTTTTGCGATACTCTAATGGAAGGC |
| *waaA* | GCGGGATCCTGGATCGCCCAAACATTACG/GCGAAGCTTAAACGCAGCCAAATCAGAGG |
| *psaA* | AAAGCGTCAAATAGCATTGGG/CAGCGATCATTAGTGTGGTAAC |
| *psaE* | CCTGTTTGTCCTGCTGATCC/GACTCATTTGCCCTCACCTC |
| YPO1635 | GCGGGATCCCCGACTCGACCGTGCTAC/GCGAAGCTTATGGCGACACTACAGGAACC |
| *phoP* | CCGGAATTCTGATGCCAGCAAAGACG/CGCGGATCCAGATGGTGACGCAACAAC |
| *fur* | CTGAGTATTTCTGTGATGCGATG/CTGACGTGGTGACACGCAGG |
| *rovA* | CGTTCGTTACTCTGCCCATC/TTGTGATTGCTCTGGTGGTAAAC |
| *rovM* | TCTGGATCCAAAGAGAACATTCAGCAGCCG/TCTAAGCTTGTTCTAAGCAGATCGAGGTCG |
